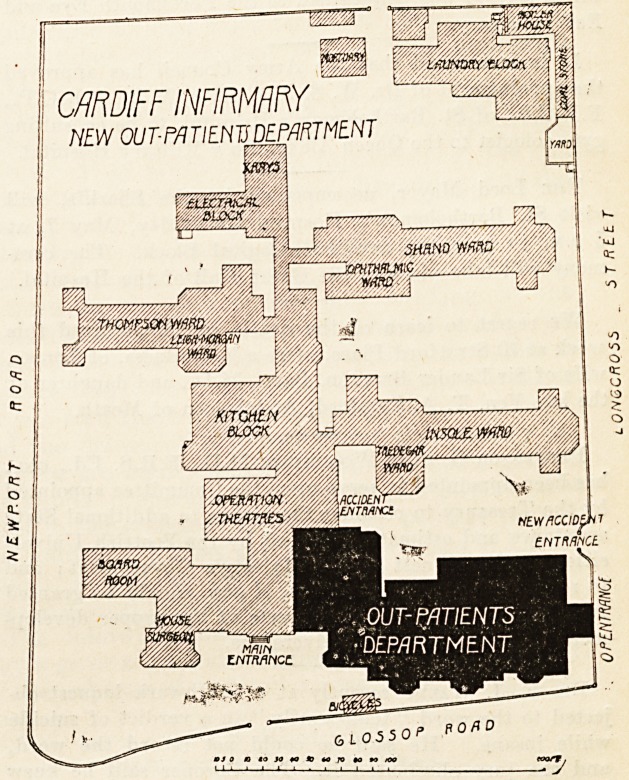# Cardiff Infirmary Extension

**Published:** 1909-04-17

**Authors:** 


### CARDIFF INFIRMARY EXTENSION.

### NEW OUT-PATIENT DEPARTMENT.

The plans show an extension and adaptation of existing buildings rather than an entirely new department, and must be judged therefore from a different standpoint than would be the case if the architect had had a free hand.

The old out-patient department, which had become far too small for the needs of the hospital, was limited to the space immediately under the nurses' old cubicles, which is indicated by the dotted lines and pillars in the waiting-hall, together with the space now occupied by the secretary's office and the dental department. The two or three consulting and examining rooms were the rooms now used as dispensary store and students' room. It is clear that the work must have been carried on under the greatest difficulty, and that the need for extension had become acute.

Ground-floor Plan.

The entrance for out-patients is at the south end of the block in Longcross Street. A wide entrance-hall gives access to a spacious lobby, on one side of which are placed the Registrar's office and porter's room. The latter commands

First-floor Plan.

the main waiting-hall by means of a glazed screen. From this lobby patients pass into the waiting-hall, which has an extreme length of nearly 100 feet and a width of 30 feet. On the south-east is the ear, nose, and throat department, consisting of a large examination-room with dark-room attached, an operation-room, and a recovery-room for each sex. Adjoining this department, but separated by the sterilising-room, is the eye department. Here is a small waiting-room, consulting-room, operation-room, refraction-room, and dark-room. On the west side of the hall at the south end is the gynæcological department, comprising a consulting-room and two examining-rooms each for two patients. Three w.c.'s for women patients separate this department from the surgeons' and physicians' rooms. These two departments are identical in form. The surgical department consists of a room for the clinical assistant, forming an ante-room to the consulting-room, two examining-rooms leading out of the clinical room and two out of the consulting-room. The physicians' rooms are arranged in a similar way, except that one of the examining-rooms is made into a dark-room of two compartments. At the north end of the block are two rooms for the dental surgeon. The w.c.'s for male patients are on the east side of the hall, and are separated by an apparently open lobby—an arrangement much superior to the corresponding offices for female patients.

On the east side of the waiting-hall is a refreshment bar, adjoining which is an exit for patients who do not require to go to the dispensary. The dispensary communicates with the main corridor of the hospital, as well as with the corridor for out-patients, but the serving-hatches shown in the latter do not now exist. Out-patients get their medicines in the waiting-room adjoining the dispensary, and pass out by the exit door adjoining. The entrance for accidents (not shown on plan) is close by this part of the building, and here are two rooms used for minor operations.

On the upper floor over the south end of the new building are fourteen cubicles for nurses, with two bath-rooms and two w.c.'s. The flat roof over the western part of the new building provides a convenient airing and recreation space for the nursing staff, and is approached by a staircase from the connecting-bridge between the main building and the old nurses' cubicles.

The plan strikes us as a compact, well-arranged straightforward method of dealing with the problem, and provides a most valuable addition to the work of the hospital.

The architect is Mr. Edwin Seward, F.R.I.B.A., who designed the original building, and who had the advantage of the collaboration of Colonel Vaughan, F.R.I.B.A., the Chairman of the House Committee.

## Figures and Tables

**Figure f1:**
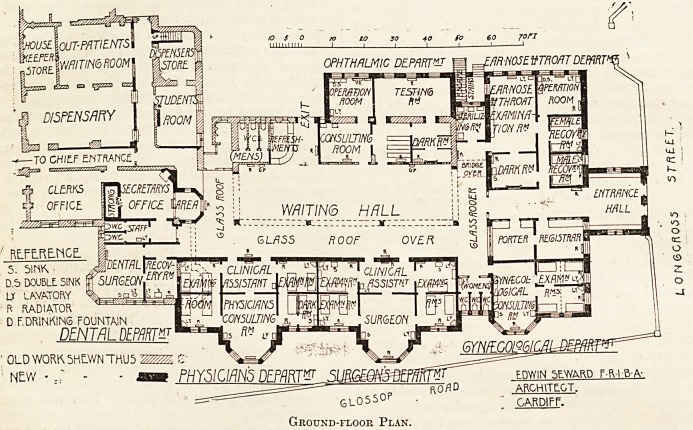


**Figure f2:**
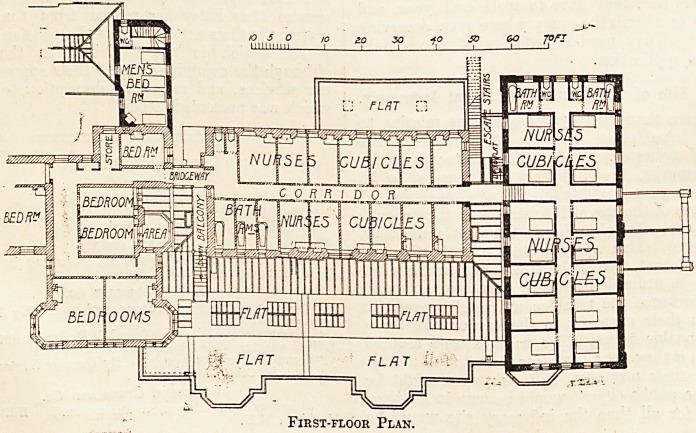


**Figure f3:**